# Healthcare professionals’ perceptions on medication communication challenges and solutions – text mining and manual content analysis - cross-sectional study

**DOI:** 10.1186/s12913-021-07227-0

**Published:** 2021-11-13

**Authors:** Tiina Syyrilä, Katri Vehviläinen-Julkunen, Marja Härkänen

**Affiliations:** 1 Department of Nursing Science, Faculty of Health Sciences, University of Eastern Finland (UEF), Yliopistonranta 1c, P.O. Box 1627, 70211 Kuopio, Finland; 2 University of Helsinki, Helsinki University Hospital (HUS), Meilahti Tower Hospital, building 1, Haartmaninkatu 4, P.O. Box 340, 00029 Helsinki, HUS Finland; 3 Kuopio University Hospital (KUH), Puijonlaaksontie 2, 70210 Kuopio, Finland

**Keywords:** Medication safety, Medication incidents, Communication, Healthcare professionals, Hospitals, Cross-sectional, Survey, Qualitative, Text mining, Content analysis

## Abstract

**Background:**

Communication challenges contribute to medication incidents in hospitals, but it is unclear how communication can be improved. The aims of this study were threefold: firstly, to describe the most common communication challenges related to medication incidents as perceived by healthcare professionals across specialized hospitals for adult patients; secondly, to consider suggestions from healthcare professionals with regard to improving medication communication; and thirdly, to explore how text mining compares to manual analysis when analyzing the free-text content of survey data.

**Methods:**

This was a cross-sectional, descriptive study. A digital survey was sent to professionals in two university hospital districts in Finland from November 1, 2019, to January 31, 2020. In total, 223 professionals answered the open-ended questions; respondents were primarily registered nurses (77.7 %), physicians (8.6 %), and pharmacists (7.3 %). Text mining and manual inductive content analysis were employed for qualitative data analysis.

**Results:**

The communication challenges were: (1) inconsistent documentation of prescribed and administered medication; (2) failure to document orally given prescriptions; (3) nurses’ unawareness of prescriptions (given outside of ward rounds) due to a lack of oral communication from the prescribers; (4) breaks in communication during care transitions to non-communicable software; (5) incomplete home medication reconciliation at admission and discharge; (6) medication lists not being updated during the inpatient period due to a lack of clarity regarding the responsible professional; and (7) work/environmental factors during medication dispensation and the receipt of verbal prescriptions.

Suggestions for communication enhancements included: (1) structured digital prescriptions; (2) guidelines and training on how to use documentation systems; (3) timely documentation of verbal prescriptions and digital documentation of administered medication; (4) communicable software within and between organizations; (5) standardized responsibilities for updating inpatients’ medication lists; (6) nomination of a responsible person for home medication reconciliation at admission and discharge; and (7) distraction-free work environment for medication communication. Text mining and manual analysis extracted similar primary results.

**Conclusions:**

Non-communicable software, non-standardized medication communication processes, lack of training on standardized documentation, and unclear responsibilities compromise medication safety in hospitals. Clarification is needed regarding interdisciplinary medication communication processes, techniques, and responsibilities. Text mining shows promise for free-text analysis.

**Supplementary Information:**

The online version contains supplementary material available at 10.1186/s12913-021-07227-0.

## Background

The National Coordinating Council for Medication Error Reporting & Prevention defines a medication incident as “any preventable event that may cause or lead to inappropriate medication use or patient harm while the medication is in the control of a healthcare professional, patient or consumer” [1]. According to a systematic review in nine countries, the cost per individual medication error ranges from a few euros up to €100,000 [2]. The World Health Organization (WHO) considers the costs of medication incidents to be substantial, both in Europe [3] and globally [4]. Such incidents may cause unnecessary harm to patients, ranging from potential but unrealized harm to severe harm or, at worst, death [5]. For healthcare professionals, the effects can range from mild psychological strain to a loss of working ability or suicidality [6]. The WHO’s third patient safety challenge, “Medication Without Harm,” aims to halve the number of medication incidents [4].

Communication issues may contribute to as many as half of the medication incidents in hospitals [7, 8]. In a systematic review by Ozavici et al. [10], 20 out of 33 studies found communication to be linked with medication discrepancies. Similarly, Keers et al. [9] concluded that 34 out of 55 studies reported communication as a contributor to medication incidents. Based on the literature, medication communication challenges can be interprofessional [11, 12], cross-organizational [12], or cross-sectoral [10, 12–14]. Communication challenges have also been studied between patients, families, and professionals [10].

The most common medication communication challenges are linked to digital and verbal communications between professionals [9, 12] as well as to situations involving care transitions [10, 12, 14, 15]. To prevent medication incidents, previous studies have recommended improving communication between nurses and physicians; between nurses and patients or family members; between hospital pharmacists and professionals [7, 12, 16, 17]; and between professionals and patients [18]. Implementing structured and digital documentation [19] and strengthening communications about compliance with guidelines have been suggested as key actions for communication improvement [11, 12].

Communication challenges have previously been investigated based on incident reports regarding general patient safety [20] or focusing on specific medication communication issues [12]. Incident reports, however, are submitted voluntarily by professionals [21]. They are estimated to cover from 0.5 % [22, 23] to somewhere under half (i.e., 45 %) of the actual incidents [24]. Audio [7] and video [25] recordings, clinical observations [19], and interviews [7, 26] have further been employed as methods for providing detailed descriptions of medication communication phenomena, though the sample sizes have been limited. To our knowledge, there is a gap in the research concerning healthcare professionals’ perceptions of medication communication challenges at the organizational and unit levels in hospitals providing specialized healthcare for adult patients. Such insight could be pivotal for improving medication safety.

The availability of digital free-text data on medication incidents is increasing, and the analysis of such data puts a time strain on research and clinical analysis [27]. New, rapid analysis methods utilizing artificial intelligence are necessary. Natural language processing (NLP) can be employed to automatically encode the semantics of free texts [28]. The NLP method can be divided into information retrieval, whereby classified data is retrieved, and information extraction, which refers to the extraction of non-classified information from free text [29]. NLP approaches can be divided into supervised [27, 29] and unsupervised text-mining methods [29, 30]. The use of supervised mining for the detection of medication incidents is currently increasing [31]. Such mining methods may combine several data sources simultaneously but require specific skills for the coding that is involved and to set the queries for data extraction [28, 29, 32]. Unsupervised, automated text mining, on the other hand, is focused on the mathematical vector spaces between used words [29]. This method aims to extract information from free-text data; when presented with a “bag of words” [33], this content is mined for lists of frequent words, related words or themes, and bunches of words that form text topics or clusters [34]. Text mining is time effective in the analysis of large data sets concerning medication incidents [27, 30, 31], but it is seldom applied to questionnaire-sized, free-text samples [35]. Only a few studies have compared text mining and manual text analysis [27]. To our knowledge, evidence on the efficacy of the text-mining method for studying medication communication challenges is lacking.

## Methods

The aims of this study were threefold: firstly, to describe the most common communication challenges related to medication incidents as perceived by healthcare professionals across specialized hospitals for adult patients; secondly, to consider healthcare professionals’ suggestions for improving medication communication; and thirdly, to explore how text mining compares to manual analysis when analyzing the free-text content of survey data.

### Design

This was a cross-sectional, descriptive study.

### Setting and description of the data

The study was conducted in 101 healthcare units that provide somatic care for adult patients throughout two Finnish hospital districts. Psychiatric and pediatric units were excluded because of the specific communication needs in these specialties. Convenience sampling [36] of the units was employed in cooperation with two collaborators from the participating organizations. All healthcare professionals in the selected units were targeted (*N*=3,892). The Raosoft sample size calculator [37] was utilized to estimate the minimum required sample size. This calculation was based on the national and organizational population sizes of the targeted professionals and a predicted minimum response rate of 10 % for the emailed survey [38, 39].

The data consisted of responses to 12 questions involving background information (e.g., unit type, position, and professional group) (see Table 1) and free-text responses in writing to two open-ended questions, which were:

**Table 1:** Demographics and background information concerning the participants (*N*=223)

Variable name of demographic or background information	Values of the responders for the open-ended question (*N*=223): “The most common medication communication challenges in one’s own clinical environment”†		Values of the responders for the open-ended question (*N*=195): “Suggestions for communication enhancements in one’s own hospital to reduce medication incidents”†	
	Valid **N** f (%)	Missing from valid **N** f (%)	Valid **N** f (%)	Missing from valid **N** f (%)
1. **Location of working unit**	*n*=223	**-**	*n*=195	-
Within the hospital	215 (96.4)		187 (95.9)	
In the outpatient services that are off the hospital site, or the responder was responsible for several locations	8 (3.6)		8 (4.1)	
2. **Unit type**	*n*=223	**-**	*n*=195	-
Inpatient unit	121 (54.3)		101 (51.8)	
Outpatient clinic or day surgery	34 (15.2)		32 (16.4)	
Intensive care unit, step down unit, operating room, or recovery room	42 (18.8)		38 (19.5)	
Elsewhere, or they were responsible for several units	26 (11.7)		24 (12.3)	
3. **Position**	*n*=221	2 (0.9)	*n*=194	1 (0.5)
Not in management position	173 (78.3)		153 (78.5)	
Manager	39 (17.7)		34 (17.4)	
Middle manager or in a chief position	9 (4.0)		7 (3.6)	
4. **Professional group**	*n*=220	3 (1.3)	*n*=193	2 (1.0)
Practical nurse	5 (2.3)		5 (2.6)	
Registered nurse	171 (77.7)		151 (77.4)	
Specialist nurse, clinical teacher, or patient safety officer	6 (2.7)		6 (3.1)	
Physician or a specialist physician	19 (8.6)		16 (8.2)	
Pharmacist	16 (7.3)		13 (6.7)	
Something else	3 (1.4)		2 (1.0)	
5. **Clinical pharmacist available in the clinic**	*n*=216	7 (3.1)	*n*=190	5 (2.6)
No or not known	41 (19.0)		37 (18.9)	
Yes	175 (81.0)		153 (80.5)	
6. **Work experience in current position in current organization**	*n*=219	4 (1.7)	*n*=192	3 (1.5)
0–5 years	108 (49.3)		95 (49.5)	
6–15 years	75 (34.2)		63 (32.8)	
≥16 years	36 (16.4)		34 (17.7)	
7. **Work experience in current type of work altogether**	*n*=220	4 (1.7)	*n*=192	3 (1.5)
0–5 years	64 (29.1)		56 (29.2)	
6–15 years	94 (42.7)		81 (42.2)	
≥16 years	62 (28.2)		55 (28.6)	
8. **Submitted a digital incident report himself/herself concerning medication error**	*n*=221	2 (0.9)	*n*=194	1 (0.5)
No	23 (10.4)		22 (11.3)	
Yes	198 (89.6)		172 (88.2)	
9. **Perception of percentage of factual medication incidents that are entered into a digital incident register**	*n*=222	1 (0.4)	*n*=193	2 (1.0)
0–30 %	89 (40.1)		60 (31.1)	
40–60 %	96 (43.2)		85 (44.0)	
70–100 %	37 (16.7)		48 (24.9)	
10. **Regularity in analysis of incident reports with staff by manager or patient safety specialist**	*n*=220	3 (1.3)	*n*=192	3 (1.5)
At least monthly	136 (61.8)		117 (60.9)	
Once or a few times per year	72 (32.7)		62 (31.9)	
Never analyzed together, or not known	12 (5.5)		13 (6.7)	
11. **Perception that sufficient information is available concerning the developments generated based on the incident reports**	*n*=222	1 (0.4)	*n*=193	2(1.0)
Not sufficient or irrelevant in this area of responsibility	99 (44.6)		81 (42.0)	
Sufficient	123 (55.4)		112 (58.0)	
12. **Years the current digital medication management system has been in use in the clinical area**	*n*=211	12 (5.4)	*n*=185	10 (5.1)
Not known	45 (21.3)		35 (17.9)	
Around one year or less	26 (12.3)		22 (11.3)	
Several years	128 (60.7)		116 (59.5)	
Old and new system are overlapping currently, while the clinical area is shifting to a new system	12 (5.7)		12 (6.2)	

† Valid = Value stating the percentage of the participants who responded (missing values were not included in the percentage calculation)

1) According to your experience, what are the main challenges in medication communication in hospitals?

2) What are your suggestions for medication communication enhancement in hospitals?

The questions (See Additional file 1) were developed for the present study based on previous results from Syyrilä et al. [12]. An expert panel (*n*=14) of healthcare professionals and patient representatives evaluated the importance and understandability of the questions. Five healthcare professionals pilot tested the survey form technically; these results were not included in the final data analysis. The same digital questionnaire included 82 additional structured (Likert-scale) questions, but the quantitative part of the study is reported in another paper (under review). Those questions assessed healthcare professionals’ perceptions of the frequency of communication challenges related to medication incidents in the hospital.

The data were collected from November 1, 2019, to January 31, 2020, using a questionnaire that was emailed to 3,892 healthcare professionals. A total of 223 (5.73 %) responses were received for the background and open-ended questions. The respondents were practical (*n*=5; 2,3 %) and registered nurses (*n*=171; 77,7 %), physicians (*n*=19; 8.6 %), pharmacists (*n*=16; 7.3 %), clinical teachers of nursing or medicine and clinical specialists (*n*=6; 2.7 %), and managers, middle-managers, or chiefs (*n*=48; 21.7 %) at all organizational levels. These healthcare professionals worked in inpatient and outpatient departments, intensive care units, operation theaters and recovery rooms, day surgeries, ambulance services, and emergency departments. Nearly half of the participants (*n*=108; 49.3 %) had worked for five years or less in their current positions in their current organizations, but the majority had worked with similar tasks from 6 to 15 years (*n*=94; 42.7 %). Participants were not asked for their sex or age for anonymity protection reasons (see Table 1).

### Data analysis

**Fig. 1:**
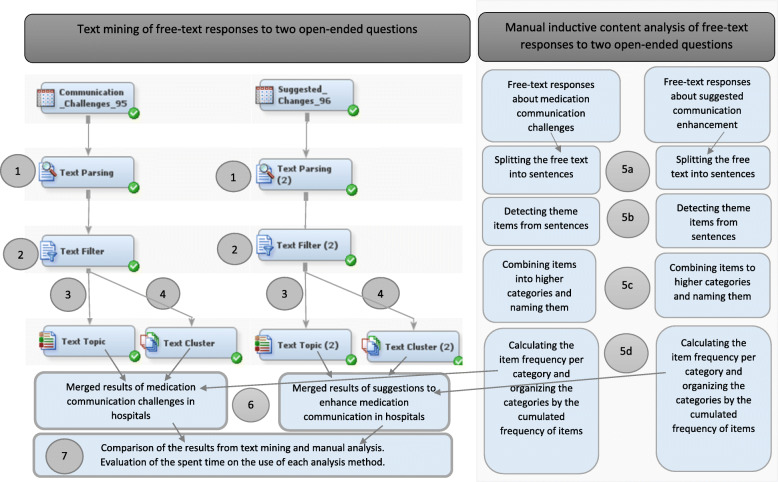
Flowchart of data analysis processes. The flowchart presents the data analysis processes of text mining and manual inductive content analysis, concerning the analysis of the free-text responses to two open-ended questions. The questions concerned medication communication challenges in a hospital and suggested improvements to medication communication. The numbers 1–7 stand for the process phases

The background variables were described using IBM SPSS version 27 software for Windows (Chicago, IL, USA). The free texts from open-ended questions were analyzed using parallel text mining (text topic extraction and text clustering) and manual inductive content analysis. The results were merged in the end [40] and the used analysis times were compared. The analysis processes of the free texts are presented in Fig. 1.

For the text-mining analysis, the free-text data were exported from the Eduix questionnaire application as .xls files and converted into .sas7bdat files using the SAS Enterprise Guide 7.1 application. The converted data files were analyzed using SAS Enterprise Miner Workstation 13.2. The free-text analysis was executed using SAS NLP methods, which consisted of the following four phases: (1) text parsing, (2) filtering, (3) extracting topics, and (4) identifying text clusters [29, 30, 34].

#### Phase 1: Text parsing

Finnish was selected as the analysis language for the parsing node. The original words and citations presented in this article were then translated to English by the first author. The default choice of stop words was used (i.e., the parts of speech to be ignored [auxiliary or modal, conjunction, determiner, interjection, numeral, particle, preposition, or pronominal] as well as the textual attributes [numbers and punctuation]). In such a way, the analysis was focused; the number of non-essential words in the text was reduced [28, 29]. Ultimately, the analyzed text from the open-ended questions comprised 7,414 words regarding challenges and 4,990 words pertaining to suggested improvements.

#### Phase 2: Text filtering

For both sets of data, the default settings were upheld for frequency limits, term weighting, and stemming (i.e., the words having a similar root word). The automated weighting of the terms was based on the terms’ frequencies in single documents and their distributions in a document collection [34]. Terms existing in a minimum of five documents were included in the analysis. However, to enable observation of the excluded terms, all terms were listed. Synonyms were manually combined using an interactive filter viewer. Synonyms and their parent terms were determined based on judgements made by the first author. Terms appearing in fewer than five documents were selected for the analysis if they were considered synonyms with a valid parent term (i.e., one that existed at least in five documents). The lists of terms, term frequencies, document frequencies, and weights of the words were verified using a “filter viewer” [34]. Example lists can be viewed in Additional files 2 and 3. The lists were submitted for the automated extraction of text topics and clusters.

The top terms regarding communication challenges was “be” (frequency 317, docs 123, weight 0.0), but it was excluded from the analysis due to its zero-weight value. In the list of the most frequently stated terms, the six most common for communication challenges were mentioned over 100 times each. The terms were: **not** (frequency 295), **medicine** (frequency 191), **patient** (frequency 185), **physician** (frequency 126), **prescription** (frequency 118), and **computer software** (frequency 101). The details of the 50 most common terms are presented in Additional file 2. Respectively, the most frequent terms for suggestions of medication communication improvement were: **medicine** (frequency 95), **patient** (frequency 88), **not** (frequency 80), **physician** (frequency 64), **need**/**should** (frequency 61), and **come** (frequency 51) (Additional file 3).

#### Phase 3: Text topic extracting

Singular value decomposition (SVD) was employed using SAS software to create a lower-dimensional space from the high-dimensional original data matrix. This was used to derive a small number of dimensions that summarized the majority of the information contained in the original input matrix [34, 41]. Interpretation of the SVD acted like a principal component analysis, as they both extract the underlying or “latent” dimensions of the information in the full data matrix [41].

A text topic node sieves the most frequent topics from the free text using a set number of correlated or uncorrelated terms, depending on the option selected by the researcher. In our study, we searched correlated words. The same words or terms could be used in one or more topics [29, 34]. The number of searched topics was set by the research team and was adjusted according to the size of the text pool [34, 42]; the smaller the sample, the lower the number of words per topic should be.

As the text sample in this study was small, the topic filtering was tested for six and ten topics. The resulting topics were compared based on the lowest term cut-off (0.101 for six topics/0.101 for ten topics), lowest document cut-off (0.12/0.118), average number of terms (19.5/20), and average number of documents (44.5/45). As the values were similar, the final number of topics was decided based on the understandability of the topic themes.

Accordingly, from the texts regarding suggested improvements, six text topics were formed. The average number of terms per topic was 18.8, and the average number of terms per document was 50.2. The extracted word combinations forming the topics were named by the research team. The citations of each topic were extracted from the free-text responses using an “interactive topic viewer.” The citations up to the cut-off point were read in full before the topics were named, but only the six automatically extracted terms per topic were used for naming the topic.

#### Phase 4: Text clustering

A “text cluster node” was utilized to extract unique clusters of similar words that appeared within one word cluster but were disjointed between different clusters (i.e., a unique cluster of similar words did not appear across multiple word clusters). The maximum number of clusters was set to ten for both data sets (i.e., communication challenges and suggestions for enhancing medication communication). The number of words per cluster was set to ten for the communication challenges and five for the suggested enhancements. The final numbers were based on different responder rates and the understandability of the cluster results with different numbers of terms (each document was used only in one cluster) [34]. The default root mean square standard measure (RMS std) was used to test the similarity of the documents in each cluster. This measure displayed the number of terms in the clusters versus the root mean squared standard deviation of the cluster. The lower the RMS std value, the more similar the documents in the cluster were [30, 34].

#### Phase 5: Manual inductive content analysis

Manual inductive content analysis of the free-text responses was conducted using an Excel table. Each response was placed in a row in the first column (5a in Fig. 1). The descriptive theme items were detected from each sentence (5b). Related items were combined to form generalized higher categories (5c) [36]. Altogether, 35 categories were written as column titles. Each observed item within a row was ascribed with the value one, which was marked in the relevant categorical column. The cumulative frequencies of each categorical column were calculated (5d). The category names were sorted according to the cumulative frequency of each column. The highest-frequency category represented the most common communication challenge. The original citations from the response texts were utilized as evidence for the named categories [36].

#### Phase 6: Merging the results from text mining and manual analysis

The results from the text mining and manual analysis were reported parallel in the same table, with one table for the challenges and another for the enhancement suggestions. The final results were formed by merging the most common medication communication challenges and, similarly, the suggested communication enhancements, as extracted from all three methods (text topics, text clustering, and manual analysis) and presenting citation evidence of the original responses.

#### Phase 7: Similarity evaluation of the results from text mining and manual analysis

The similarity of the extracted results from the text topics, text clustering, and manual analysis methods was evaluated. Comparisons were completed separately for both of the open-ended questions. The time spent on each analysis method was compared.

## Results

### Categories of medication communication challenges according to each method

**Fig. 2:**
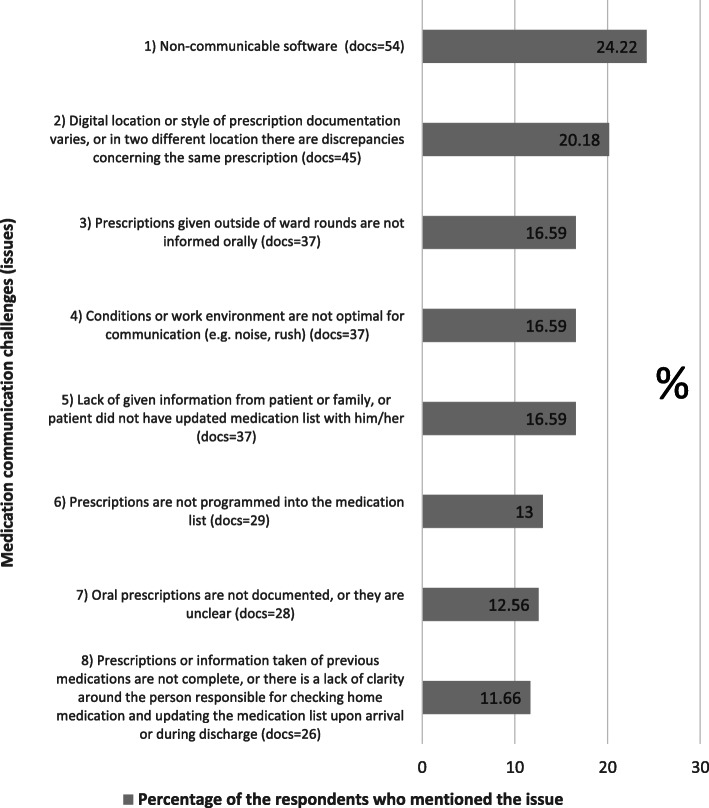
Eight most common medication communication challenges based on the manual content analysis (*N*=223)

Text mining identified six text topics (Table 2) and seven text clusters (Table 3). Respectively, through manual content analysis, the eight most common communication challenges from a total of 35 detected challenge categories were related to the results of the text mining (Fig. 2). The main challenges were similar, but the ranking varied. The most common challenges involved communication about administered medication (topic filtering), home medication reconciliation (text clustering), and non-communicable software (manual analysis).

**Table 2:** Text topics of medication communication challenges in hospitals: an analysis of the responses to open-ended questions (*N*=223)

Topic number	Filtered words/terms forming the topic † (The original terms in Finnish are presented below in parentheses)	Term cut-off	Document cut-off	Number of terms	Number of documents including the topic	Highest weight for one word in the word combination of the topic	Topic names given by the authors (Numbered according to the number of documents including the topic)
1	documenting + physician + verbal + prescription + to document (kirjaaminen + lääkäri + suullinen + määräys + kirjata)	0.102	0.179	18	54	0.452	2. Discrepancies between written notes and verbally given prescriptions
2	ward round + prescriptions given outside of ward round + new + outside + stay (lääkärinkierto + lääkärinkierron ulkopuoli tehtävä lääke-määräykset + uusi + ulkopuoli + jäädä)	0.103	0.135	20	36	0.309	5. Failure to notice prescriptions documented outside the ward rounds
3	medicine + patient + give + all + medication care (lääke + potilas + antaa + kaikki + lääkehoito)	0.103	0.153	23	57	0.38	1. Ambiguity around whether the patient has been given all their medication
4	medication list + list + patient + being + to set (lääkelista + lista + potilas + oleva + laittaa)	0.103	0.12	27	24	0.399	6. Challenges in updating the medication list for the patient
5	transfer + other + unit + gap + software (siirto + toinen + yksikkö + väli + tietojärjestelmä)	0.101	0.175	13	46	0.505	4. Non-communicable software during the transition between units
6	software + challenge + usage + different/diverse + new (tietojärjestelmä + haaste + käyttö + eri + uusi)	0.102	0.162	18	50	0.482	3. Challenges related to the use of diverse/new software

† The original words and terms in Finnish were filtered by SAS Enterprise text miner 13.2

**Table 3:** Text clusters of the medication communication challenges in hospitals: based on responses to open-ended questions (*N*=223)

Cluster ID	Terms of the cluster † (The original terms in Finnish are presented in parentheses)	Cluster frequency (f)	Cluster percentage (%)	Root mean square standard (RMS std)	Cluster name formed by the authors
1	Terms of this cluster are not included in the analysis	125	36	0.0	Not applicable
2	**+ medication list + to know + several + patient + wrong + drag + get + own + home medication** (+ lääkelista + tietää + lääkitys + usea + potilas + väärä + lääke + saada + oma + kotilääkitys)	56	16	0.147	1) Challenges around the home medication reconciliation for a patient
3	**+ another + transition + between + unit + report + software + different + own + usage + hospital** (+ toinen + siirto + väli + yksikkö + raportti + tietojärjestelmä + eri + oma + käyttö + sairaala)	33	10	0.139	3) Communication challenges related to the care transition and non-communicable software
4	**+ physicians’ ward round + documenting + prescription given outside of ward round + after + order by physician + medication prescription + physician + unclear + nurse + stay/leave** (+ lääkärinkierto + kirjaaminen + lääkärinkierron ulkopuoli tehtävä lääkemääräykset + jälki + määräys + lääkemääräys + lääkäri + epäselvä + hoitaja + jäädä)	44	13	0.143	2) Lack of clarity around the medication prescriptions that have been given outside the ward rounds
5	**+ checking + challenge + usage + communication + change/amendment + unclear + give + software + home medication + rush** (+ tarkistaminen + haaste + käyttö + kommunikaatio + muutos + epäselvä + antaa + tietojärjestelmä + kotilääkitys + kiire)	28	8	0.152	4) Challenges associated with documented medication changes and the checking of these in the digital system
6	**+ document + go + uncomplete + have time + cause/lead to + software + verbal + wrong + use + several** (+ kirjata + mennä + puutteellinen + ehtiä + aiheuttaa + tietojärjestelmä + suullinen + väärä + käyttää + usea)	19	6	0.139	6) Challenges associated with the uncompleted documentation of verbal prescriptions
7	**+ medication care + care + digital + part + dispense/distribute + same + noise + use always + time** (+ lääkehoito + hoito + sähköinen + osa + jakaa + sama + melu + käyttää aina + aika)	27	8	0.146	5) Challenges associated with medication dispensing and digital information
8	**+ interruption + colleague + constant + medication dispensing + time + noise + home medication + amendment of medication + see + dose** (+ keskeytys + kollega + jatkuva + lääkkeenjako + aika + melu + kotilääkitys + lääkemuutos + nähdä + annos)	12	3	0.132	7) Noise and interruptions during the medication dispensation that affect the correct determination of the dose

† The original words and terms in Finnish were filtered by SAS Enterprise text miner 13.2

### Merged medication communication challenges

The merged results from all three methods (Table 4) described the following challenges:

**Table 4:** Medication communication challenges in hospitals: the results from text mining and manual analysis (*N*=223)

Medication communication challenges		
**Text mining**		**Manual analysis**
**Text topic filtering**	**Text clustering**	**Inductive content analysis**
1) Ambiguity around whether the patient has been given all of their medication (docs=57)	1) Challenges concerning patient home medication reconciliation (f=56)	1) Non-communicable software (docs=54)
2) Discrepancies between the written notes and orally given prescriptions (docs=54)	2) Lack of clarity on medical prescriptions given outside the ward rounds (f=44)	2) Location or style of prescription documentation varies, or there are discrepancies in two different locations concerning the same prescription (docs=45)
3) Challenges related to the use of diverse/new software (docs=50)	3) Communication challenges related to the care transition and non-communicable software (f=33)	3) Prescriptions that are given outside the ward rounds are not informed orally (docs=37)
4) Non-communicable software during the transition between units (docs=46)	4) Challenges associated with documented medication changes and checking these in digital systems (f=28)	4) Conditions or work environment are not optimal for communication (e.g., noise, rush) (docs=37)
5) Failure to notice prescriptions documented outside the ward rounds (docs=36)	5) Challenges associated with medication dispensing and digital information (f=27)	5) Lack of given information from the patient or family, or the patient did not have an updated medication list with him/her (docs=37)
6) Challenges in updating patient medication lists (docs=24)	6) Challenges associated with incomplete documentation of oral prescriptions (f=19)	6) Prescriptions are not programmed into the medication list (docs=29)
- The mining program did not generate its own topic for conditions. However, conditions-related words and phrases (e.g., rush, be in time) appeared in three topics of the six	7) Noise and interruptions during the medication administration that affect the ability to determine the dose (f=12)	7) Oral prescriptions are not documented, or they are unclear (docs=28)
		8) Lack of clarity regarding the person responsible for checking home medication and updating the medication list at arrival or during discharge, or the prescriptions or information taken of previous medications are not complete (docs=26)

1) Inconsistent documentation of prescribed and administered medication.

*“… colleagues have diverse ways (of documentation). Even if the organisation provides guidance for medication documentation protocol, it is not followed…”* [Respondent (R)220]

*“The only real challenge is that the right prescriptions are documented in the right location. Meaning, that it should be done by a physician to the (digital) ‘Medication list.’”* [R16].

*“…It is not enough that the medicine is on the list, I want to know if it has been administered or not. Some patients have the medication on the list, but the patient has not been given the medicine. Department-x is annoying regarding documentation: documentation is not completed and the nursing staff do not answer the phone. Shift changes increase the risk that a patient is given the same medication repeatedly or some medication is omitted totally.”* [R6].

2) Undocumented orally given prescriptions.

*“Physicians often give oral prescriptions instead of clearly documenting them digitally. Orally given prescription can be misheard or misunderstood.”* [R78].

3) Nurses’ failure to notice prescriptions (given outside of the ward rounds) due to a lack of verbal communication from the prescribers.

*“There is no alerting signal in a patient’s record about new prescriptions written by the physician. Amendments have been noticed with several hours’ delay or left non-executed, because they (the prescriptions) have been given by an on-call physician outside of the ward rounds and those (amendments) haven’t been discussed, even during the ward rounds.”* [R166].

4) Communication breaks during a care transition due to non-communicable software or another non-communicable method.

*“… health centres have their own software, so, in the worst case, the medication lists—in our hospital, in a health centre, from the physician’s referral, and with the (national) prescription centre—are conflicting.”* [R209].

5) Incomplete home medication reconciliation at admission and at discharge due to a lack of clarity regarding the responsible professional.

*“A major communication challenge is related to the fact that the digital patient records and the latest information on medication are not available. In addition, there is an unclear division of tasks… who is responsible for home medication reconciliation?”* [R118].

*“Many patients visit in inpatient and outpatient departments where the medication lists have not been updated digitally after discharge.”* [R209].

6) Medication lists not being updated during inpatient periods due to a lack of clarity regarding the responsible professional.

*“… during patient transition, e.g., from the ICU/step-down unit to the inpatient department. The medication list is often unclear, or it is totally missing, or the medication is different on the medication list compared to the notes written by the physician.”* [R143].

7) Communication challenges during medication dispensation and the receipt of verbal prescriptions due to work/environmental factors.

*“A rush compromises everything. The medication administration room is a central location of noise. The physician gives prescriptions using information relating to the patient’s bed location or diagnoses, and even talks towards a computer. It is challenging to know to whom he/she is talking to and about which patient.”* [R194].

### Categories of suggestions for enhancing medication communication according to each method

Text mining determined five text topics (Table 5) and six text clusters (Table 6) regarding the improvement of medication communication. According to the manual inductive content analysis, there were 35 total suggestion categories for enhancing medication communication in hospitals, nine of which were related to the text mining results. The list of the related suggestions formed through manual analysis is presented in Table 7. The most common suggestions for communication enhancement involved discussions about new prescriptions (text topics) and medication lists (text clustering) between nurses and physicians and securing communicable software within and between organizations (manual analysis).

**Table 5:** Text topics of suggested medication communication enhancements: based on an analysis of open-ended questions (*N*=195)

Topic number	Filtered words/terms forming the topic † (The original terms in Finnish are presented below in parentheses)	Term cut-off	Document cut-off	Number of terms	Number of documents including the topic	Highest weight for one word in the word combination of the topic	Topic names given by the authors
1	prescription + software + clear + own + also (määräys + tietojärjestelmä + selkeä + oma + myös)	0.121	0.127	19	54	0.403	2. Clear prescriptions in the software system
2	Not + guideline + medication care + get + know (ei + ohje + lääkehoito + saada + tietää)	0.12	0.131	18	36	0.32	4. Knowledge of clear medication care guidelines
3	Nurse + physician + do + prescription + come (hoitaja + lääkäri + tehdä + lääkemääräys + tulla)	0.12	0.163	17	57	0.359	1. Information transfer concerning new prescriptions between the physicians and nurses
4	Patient recording system + same + medication + software system + all (potilastietojärjestelmä + sama + lääkitys + tietojärjestelmä + kaikki)	0.12	0.148	20	24	0.417	5. Communicable medication management software systems
5	Should + medicine + patient + medication list + medication (pitää + lääke + potilas + lääkelista + lääkitys)	0.118	0.177	20	46	0.341	3. Should update the medication list of the patient

† The original words and terms in Finnish were filtered by SAS Enterprise text miner 13.2

**Table 6:** Text clusters of suggested hospital medication communication enhancement: based on an analysis of open-ended questions (*N*=195)

Cluster ID	Terms included in the cluster † (The original terms in Finnish are presented below in parentheses)	Cluster frequency	Cluster percentage	RMS std (Root mean square standard)	Topic name formed by the authors (Numbered 1–6, starting from the most frequent)
1	terms excluded from analysis	158	46	0	Not applicable
2	+ to know + very soon + communication + guideline + information (+ tietää + kohta + kommunikaatio + ohje + tieto)	29	8	0.17	3) Securing communications about guidelines and related information
3	+ example + part + work + another + do (+ esimerkki + osa + työ + toinen + tehdä)	11	3	0.15	6) Securing the necessary skills for when conducting the tasks of another member of the team
4	+ software + all + system + clear + prescription/order (+ tietojärjestelmä + kaikki + järjestelmä + selkeä + määräys)	45	13	0.16	2) Giving clear prescriptions in the digital system
5	+ patient health record + same + transfer + another + give (+ potilastietojärjestelmä + sama + siirtyä + toinen + anna)	12	3	0.14	5) Using the same patient health record system during the transition
6	+ as/so + education/training + reporting + good + clarified (+ niin + koulutus + raportointi + hyvä + selkeä)	24	7	0.17	4) Undertaking training for clear reporting
7	+ nurse + physician + come + medication + medication list (+ hoitaja + lääkäri + tulla + lääke + lääkelista)	65	19	0.16	1) Enhancing the communications between nurses and physicians about medication lists and medicines

† The original words and terms in Finnish were filtered by SAS Enterprise text miner 13.2

**Table 7:** Healthcare professionals’ suggestions for improving medication communication: the results from text mining and manual analysis (*N*=195)

Suggested medication communication enhancement: analyzed responses to open-ended questions		
**Text mining**		**Manual analysis**
**Text topic**	**Text clustering**	**Inductive content analysis**
1) Information transfer concerning new prescriptions between the physicians and nurses (docs=57)	1) Enhancing the communication between nurses and physicians about medication lists and medicines (docs=65)	1) Communicable software within and between organizations (docs=29)
2) Clear prescriptions in the software (docs=54)	2) Giving clear prescriptions in the digital system (docs=45)	2) Standardized documentation places and styles, not allowing for any individual variance (docs =28)
3) Should update the medication list of the patient (docs=46)	3) Securing communications about the guidelines and related information (docs=29)	3) Clarified prescriptions (i.e., what has been changed and why, the starting time, the stop day, what is to be observed and evaluated) (docs =23)
4) Getting to know clear guidelines (docs=36)	4) Undertaking training for clear reporting (docs=24)	4) Prescriptions are documented straight to the medication list and should be completed by physicians (docs =22)
5) Communicable medication management software systems (docs=24)	5) Using the same patient health record systems during the transition (docs=12)	5) Standardized responsibilities for the tasks in medication documentation (e.g., clarify who is responsible for checking the home medication of patients) (docs=19)
-	6) Securing the necessary skills when conducting the tasks of another member of the team (docs=11)	6) Calm conditions for communication during the medication administration (docs=19)
-	-	7) Documentation of given doses in a timely manner (docs=18)
-	-	8) Training on the software, responsibilities, medication process, and medicines (docs=16)
-	-	9) Verbal information about the prescriptions given outside the ward round (docs=16)

### Merged suggestions for communication enhancement

The merged results from the three analysis methods suggested seven ideas for the enhancement of medication communication based on text mining and manual analysis (Table 7):

1) Structured and digital prescriptions.

*“… there should be only one location—currently, it varies, e.g., in the digital medication chart, physicians’ daily orders, decursus—where we document medication…”* [R11].

*“All prescriptions should be done in writing according to agreed protocol.”* [R156].

*“The physician documents prescriptions by himself/herself. The patient’s medication list in one location is updated. Now, when the patient arrives for an operation, the medication list might have been printed 2–4 days ago, but only the digital list is updated.”* [R7].

2) Guidelines and training on using the documentation systems, a documentation style, where to document, and who is responsible for documentation.

*“… the whole organisation should have consistent practices, for which all staff should be trained, and which are systematically implemented. Also, attitude training is needed. Often, when a patient arrives to our department, they have omitted medication, because (the staff) in the sending department assumed that the receiving department would take care of the medication.”* [R169].

3) The timely documentation of verbal prescriptions and digital documentation of administered medication.

*“Medication, communicated prescriptions, and amendments are always prescribed in writing and orally.”* [R54].

*“… Another thing is that medication is documented in real time…”* [R164].

*“… the physician is responsible for medication care… a prescription is made in writing and any suspended medication is marked; when a new medication is described, the current medication is checked; for a periodic medication, the finishing date is stated, so it is clear that the medication is not continuous; the starting time of the medication (clock time, too) should be included with prescription; in addition, if acute, urgent amendments are needed, then oral notice should be given as prescribing only to the ‘lati’ (the digital system) means the information would not reach the nurses in time; patient´s allergies should be observed when prescribing medication.”* [R112].

4) Communicable software across and between organizations.

*“One medication management software, which is used by everyone with confidence.”* [R155].

*“… everyone, everywhere in Finland, should have the same digital patient record software from primary care to specialised healthcare.”* [R151].

5) Standardized responsibilities for updating inpatients’ medication lists.

*“Someone should write the documentation in a digital system when the patient arrives. Very simple. Then, strict rules are needed so that everyone documents them (the medications) in a digital form within the hospital when amendments are prescribed. Everything has to be found in one single location, which is the medication list in the computer program.”* [R22].

6) A responsible person nominated for home medication reconciliation at the points of admission and discharge and for documenting any changes in a digital format.

*“Who is to document patients’ home medication into a digital medication list? The physician may dictate them (medication) but does not have the time to write them. It is a big challenge. And who will remove many-years-old medication, which is not in use anymore, from the medication list? In the accident and emergency department, it takes easily one hour for a physician to remove them, if the physician conducts it by himself/herself.”* [R27].

*“Medication lists should be kept updated, even if it might take the majority of the allocated time of the appointment.”* [R75].

7) Distraction-free conditions and work environment for medication communication.

*“Respectful, non-distracting surroundings for the medication administrator, with limited distractions and rushing, will result in clear prescriptions and confidence in using digital systems. Clear consultation possibilities regarding medication care.”* [R67].

### Comparison of text mining and manual analysis methods

The most common contents between the filtered text topics, clusters, and categories formed by manual analysis were found to be similar, although the wording, rankings, and depth of detail varied between the diverse methods.

Manual analysis extracted the most detailed information about the challenges and suggestions, followed by text topic extractions, while text clustering merely gave categorical information. The text mining methods concentrated the texts without necessarily requiring a person to handle any of the citations manually. The text topic feature also came with an option to retrieve the original citations linked to specific text topics, if required.

Text mining took less time than the manual analysis methods. In the text mining process, the pre-analysis tokenization took two to three hours per open-ended question. Filtering all text topics or all text clusters took from 5.16 to 14.36 s in total. The authors then spent a few hours naming the automatically formed topics and clusters. In comparison, the manual inductive content analysis of the same texts took days.

## Discussion

To our knowledge, this is the first study to describe healthcare professionals’ views of medication communication challenges and their suggestions for improving communication in adult nursing settings in hospitals, covering all organizational levels and settings and numerous specialties. By merging the results from text mining and manual content analysis, we extracted seven communication challenges and seven suggestions for improving medication communication. The text mining method proved promising for free-text analysis, and further testing is recommended.

The most common medication communication challenges involved incomplete or missing digital documentation of administered medication or a lack of digital documentation regarding verbal prescriptions. These results mirror those from previous studies [12, 19]. However, as Myers et al. [43] found in their observational cohort study, the lack of verbal information regarding documented prescriptions given outside the ward rounds was an equally common challenge. Other communication barriers found in the current study involved non-communicable software and inconsistent digital documentation styles or documentation locations in a digital system. Inconsistency resulted from a lack of instructions or training about the use of digital systems or either missing or unclear documentation standards, findings that are supported by a previous study [44]. Patients’ home medication reconciliation and related documentation during admission, at discharge, and during inpatient periods were major challenges due to the unclear division of responsibilities. Improvement in reconciliation rates is pivotal for the future; Meguerditchian et al. [45] found that more than half of medication adverse events are caused by discrepancies in patients’ medication information.

The most common suggestions for improving medication communication included the standardization and training of prescription processes and digital documentation. In addition, systematic oral reporting of written prescriptions was recommended. Echoing the previous literature [11, 12, 45, 46], the professionals in the present study suggested a clarified division of responsibilities regarding medication reconciliation and documentation. However, methods of dividing these responsibilities varied across responses in the study. Thus, it seems that some degree of interdisciplinary cooperation is necessary before the division of responsibilities can become clear. Professionals’ conflicting views about the division of responsibilities also seems to be a long-standing challenge internationally [47]. New actions are needed for securing medication reconciliation. According to previous literature, management and leadership must successfully implement such interventions at the unit level [44, 48]. Furthermore, the current study highlighted the need for distraction-free conditions and work environments for enhancing medication-related communication.

Contrary to recommendations in the literature [7, 15, 17, 46], the respondents in the current study seldom proposed patient involvement as a communication enhancement target. The professionals merely highlighted the communication barriers among themselves; they described mostly technical or task-division solutions. Similarly, in their focus group study, Verhaegh et al. [49] observed that healthcare professionals do not actively encourage patients to participate in team communication.

In the current study, self-efficacy of professionals and diversity of interprofessional communication styles were rarely proposed as communication barriers, which is contractionary to the evidence in previous literature [50]. The reason for this result might be typical cultural differences.

Surprisingly, in the present study, professionals rarely stated that pharmacists in their departments played a role in advancing medication-related communication. Similarly, Berdot et al.’s [51] meta-analysis presented weak evidence of the advantage of using pharmacists. Controversially, in many studies, such as in a meta-analysis [52] and in an analysis of computerized physician order entries [53, 54], pharmacists were found to increase medication safety. In the current study, the reason for the low number of suggestions proposing strengthening the role of department pharmacists may have been because more than three quarters reported already having department pharmacists available.

When the manual analysis method was compared to text mining, text topics and text cluster extraction were found to be time-saving methods. The text-topics method gave wider descriptive information than the clustering method and provided the opportunity to relate the topics back to original citations as evidence. Text mining appears a promising and scalable method for free-text analysis from samples that comprise a couple of hundred reports up to the traditionally preferred large data sets [8, 27, 35].

### Implications for practice

The present study’s results concerning communication challenges can be applied to frame standardization actions for medication-related communication in hospitals. Text mining presents a potential opportunity to develop a clinical tool for extracting categorized free-text data [32, 33, 55] for managerial decision making.

### Implications for research

The communication challenges and suggestions for communication improvement could be applied for intervention studies to improve medication communication in hospitals. It will be useful to perform effectiveness research on interventions applied for the improvement of medication communication. Further testing of text-mining methods is recommended, not only with diverse sample sizes and in diverse settings regarding medication communication but also regarding other patient safety topics.

### Strengths and limitations

The present study’s first author presented the study plan to nurse managers and physician leaders, visited all units during the data collection period, sent two reminder emails to the managers, and arranged a motivational raffle to remind everyone about the study; still, the study’s response rate was low, as it often is with digital questionnaires [38, 39]. The questionnaire was long and, as such, may have been overwhelming for the respondents. However, though it may have been small, the final sample represented all unit types, professional groups, and organizational levels, reflecting the variety of professionals working in specialized healthcare in Finland. Even if the results are supported by literature, caution is advised when making any generalizations to the specialized healthcare of adult patients due to the sample size and descriptive nature of the qualitative research [36].

In qualitative research, validity is described as credibility and reliability as dependability [56, 57]. Credibility of the results of this study was enhanced by testing the two text-mining methods and the manual method simultaneously [36]. The dependability was strengthened by the numerical measures of the mining and manual analysis, while the credibility was strengthened by the original citations that were included from the free-text responses. The transferability of the results of this study [36, 57] is supported by the detailed descriptions of the analysis process [36].

Text-mining analysis was conducted prior to manual analysis, which may have introduced minor bias into the results. The analysis process of text mining and manual analysis included decisions that were based on the researchers’ individual judgments, which increases the risk of bias. However, the results were checked by all authors to reach a consensus. The wording was amended until it was mutually agreed upon, which somewhat limits the risk of bias [36]. Furthermore, the researchers adhered to the following EQUATOR Standards for Reporting Qualitative Research: Consolidated criteria for reporting qualitative research (COREQ).

## Conclusions

Non-communicable software, non**-**standardized medication communication processes, a lack of training on handling standardized documentation, and an unclear distribution of responsibilities compromise medication-related safety in hospitals. Clarification is necessary regarding interdisciplinary medication communication processes, documentation techniques, and responsibilities in medication-related communication. Text mining appears promising for free-text analysis.

## Supplementary information

Additional file 1 “Digital questionnaire translated from Finnish to English”. Description of data: Digital questionnaire form of current cross-sectional study named “Healthcare professionals’ perceptions on medication communication challenges and solutions”, which was conducted for text mining and manual content analysis.

Additional file 2 “Fifty most common terms linked to medication communication challenges in hospitals (*N*=223)”. Description of data: Terms of medication communication challenges, which were extracted from free text of study data using IBM SPSS version 27 software for Windows (Chicago, IL, USA) in text filtering phase.

Additional file 3 “Fifty most common terms linked to suggested medication communication enhancement in hospitals (*N*=195)”. Description of data: Medication communication enhancement terms extracted from free text of study data using IBM SPSS version 27 software for Windows (Chicago, IL, USA) in text filtering phase.

## Data Availability

The data is not publicly available, based on the decision given by the University of Eastern Finland Committee on Research Ethics (Reference number 13/2019 August 28, 2019). De-identified dataset may be made available upon reasonable request of the corresponding author.

## Abbreviations

NLP: Natural language processing
RMS std: Root mean square standard measure
SVD: Singular value decomposition
WHO: World Health Organization
