# Supplementary material for: Healthcare professionals’ perceptions on medication communication challenges and solutions – text mining and manual content analysis - cross-sectional study
Source: BMC Health Serv Res. 2021 Nov 13;21:1226. doi: 10.1186/s12913-021-07227-0 (PMC8590289; doi:10.1186/s12913-021-07227-0)
Supplement: Supplementary file 1 — “Digital questionnaire translated from Finnish to English”. Description of data: Digital questionnaire form of current cross-sectional study named “Healthcare professionals’ perceptions on medication communication challenges and solutions”, which was conducted for text mining and manual content analysis. [file 12913_2021_7227_MOESM1_ESM.docx]

Additional file 1. “Digital questionnaire translated from Finnish to English”

| **COMMUNICATION RELATED TO MEDICATION INCIDENTS – QUESTIONNAIRE FOR HEALTH CARE PROFESSIONALS** | |
| --- | --- |
| **□Your informed consent to participate in the study:** Yes, I have read the information about the study and about handling the data, and the text about participating to the study. I confirm that I will participate in the study. | |
| **□Your informed consent that the data can be used in future studies.** Yes, I will give my permission to use the data in studies of medication safety by the same researcher group, within the same restrictions as in this study (+ the appropriate ethical evaluation is conducted separately for each study). | |
| **BACKGROUND INFORMATION** | |
| 1. **Location of your working unit**   This was automatically visible from the digital drop-down list before the participant selected his/her answer from the list.  Not answered  Within the hospital  Within hospital building, but it is outpatient services.  In the outpatient services that are off the hospital  Health center  Nursing home or day center  I am responsible for several locations | |
| 1. **Your working unit type** | |
| Not answered |  |
| Inpatient unit |  |
| Outpatient clinic or day surgery |  |
| Intensive care unit, step down unit, operating room, or recovery room | |
| Elsewhere |  |
| I am responsible for several units |  |
| 1. **Your position** (?) = Behind a question mark there was the following additional information: “The results will be reported on national level only and groups having less than five respondents are merged with another group when reported”) | |
| Not answered |  |
| Not in management position |  |
| Manager |  |
| Middle manager |  |
| Chief position |  |
| 1. **Your professional group** (?) = Behind a question mark there was following additional information: “The results will be reported on national level only and groups having less than five respondents are merged with another group when reported” | |
| Practical nurse |  |
| Registered nurse |  |
| Specialist nurse |  |
| Physician |  |
| Specialist physician |  |
| Pharmacist |  |
| Clinical teacher (nursing) |  |
| Clinical teacher (medical) |  |
| Patient safety officer (within one unit) |  |
| Patient safety/quality lead |  |
| Something else |  |
| 1. **Is there clinical pharmacist available in your working unit/ in your responsibility area?** (?)= Behind the question mark there was the following explanation: By responsibility area is meant for example manager being responsible for one or several units or temporary staff working for one or several units) | |
| Not answered |  |
| No |  |
| Yes |  |
| I don´t know |  |
| 1. **Your work experience in current position in current organization**(Write years as numbers rounding to the nearest year) | |
| 1. **Your work experience in current type of work altogether** (Write years as numbers rounding to the nearest year) | |
| 1. **Have you submitted a digital incident report yourself concerning medication error?** | |
| Not answered |  |
| No |  |
| Yes |  |
| 1. **What is your perception of percentage of factual medication incidents that are entered into a digital incident register in your responsibility area?** | |
| 0% |  |
| 10% |  |
| 20% |  |
| 30% |  |
| 40% |  |
| 50% |  |
| 60% |  |
| 70% |  |
| 80% |  |
| 90% |  |
| 100% |  |
| 1. **In your own working unit or in the area of your responsibility: What is regularity in analysis of incident reports with staff by your manager or patient safety specialist?** | |
| Not answered |  |
| I do not know |  |
| Daily |  |
| Weekly |  |
| Monthly |  |
| Few times per year |  |
| Once a year |  |
| Never analyzed together |  |
| 1. **What is your perception: Is there sufficient information available concerning the developments generated based on the incident reports?** | |
| Not answered | |
| Not | |
| Yes | |
| It does not concern my responsibility area | |
| 1. **How many years has the current digital medication management system been in use in your clinical area?** | |
| Not answered |  |
| I don´t know |  |
| Around one year or less |  |
| Several years |  |
| Old and new system are overlapping currently, while the clinical area is shifting to a new system | |
| **OPEN-ENDED QUESTIONS** | |
| 1. **According to your experience, which are the main challenges in medication communication in hospitals?**   *unlimited space for writing the answer* | |
| 1. **What are your suggestions for medication communication enhancement in hospitals?** | |
| *unlimited space for writing the answer* | |
