# Supplementary material for: Healthcare professionals’ perceptions on medication communication challenges and solutions – text mining and manual content analysis - cross-sectional study
Source: BMC Health Serv Res. 2021 Nov 13;21:1226. doi: 10.1186/s12913-021-07227-0 (PMC8590289; doi:10.1186/s12913-021-07227-0)
Supplement: Supplementary file 2 — “Fifty most common terms linked to medication communication challenges in hospitals (N=223)”. Description of data: Terms of medication communication challenges, which were extracted from free text of study data using IBM SPSS version 27 software for Windows (Chicago, IL, USA) in text filtering phase. [file 12913_2021_7227_MOESM2_ESM.docx]

Additional file 2

Fifty most common terms linked to medication communication challenges in hospitals (N=223)

| **Term**  (original Finnish term in parentheses) | **Role** | **Status** | **Weight** | **Imported Frequency** | **Frequency** | **Number of imported Docs** | **#Docs** | **Rank** | **Parent/child Status** | **Parent ID** |
| --- | --- | --- | --- | --- | --- | --- | --- | --- | --- | --- |
| +not  (+ ei) | Noun | Keep | 0.128 | 212.0 | 295.0 | 117.0 | 137.0 | 1.0 | + | 11.0 |
| +be  (+ olla) | Verb | Drop | 0.0 | 317.0 | 317.0 | 123.0 | 123.0 | 2.0 | + | 68.0 |
| +medicine  (+ lääke) | Noun | Keep | 0.208 | 184.0 | 191.0 | 90.0 | 92.0 | 3.0 | + | 484.0 |
| +patient  (+ potilas) | Noun | Keep | 0.216 | 182.0 | 185.0 | 89.0 | 90.0 | 4.0 | + | 27.0 |
| +physician  (+ lääkäri) | Noun | Keep | 0.199 | 103.0 | 126.0 | 71.0 | 83.0 | 5.0 | + | 345.0 |
| +prescription/order  (+ määräys) | Noun | Keep | 0.218 | 64.0 | 118.0 | 50.0 | 78.0 | 6.0 | + | 811.0 |
| +ICT system  (+ tietojärjestelmä) | Noun | Keep | 0.252 | 35.0 | 101.0 | 30.0 | 63.0 | 7.0 | + | 1226.0 |
| +nurse  (+ hoitaja) | Noun | Keep | 0.270 | 72.0 | 85.0 | 52.0 | 57.0 | 8.0 | + | 239.0 |
| +unit  (+ yksikkö) | Noun | Keep | 0.303 | 17.0 | 59.0 | 16.0 | 46.0 | 9.0 | + | 2037.0 |
| +medication list  (+ lääkelista) | Noun | Keep | 0.329 | 73.0 | 73.0 | 44.0 | 44.0 | 10.0 | + | 22.0 |
| +information  (+ tieto) | Noun | Keep | 0.309 | 53.0 | 54.0 | 43.0 | 44.0 | 10.0 | + | 84.0 |
| +for example  (+ esim) | Noun | Drop | 0.0 | 52.0 | 53.0 | 43.0 | 43.0 | 12.0 | + | 208.0 |
| +transfer  (+ siirto) | Noun | Keep | 0.337 | 4.0 | 62.0 | 3.0 | 41.0 | 13.0 | + | 2525.0 |
| +oral  (+ suullinen) | Adj *) | Keep | 0.323 | 27.0 | 46.0 | 27.0 | 40.0 | 14.0 | + | 456.0 |
| +another  (+ toinen) | Noun | Keep | 0.338 | 25.0 | 52.0 | 21.0 | 39.0 | 15.0 | + | 649.0 |
| +medication prescription  (+ lääkemääräys) | Noun | Keep | 0.330 | 24.0 | 45.0 | 23.0 | 39.0 | 15.0 | + | 1059.0 |
| +come  (+ tulla) | Verb | Keep | 0.345 | 51.0 | 51.0 | 39.0 | 39.0 | 15.0 | + | 1135.0 |
| +medication  (+ lääkitys) | Noun | Keep | 0.342 | 48.0 | 50.0 | 38.0 | 38.0 | 18.0 | + | 309.0 |
| +give  (+ antaa) | Verb | Keep | 0.350 | 44.0 | 50.0 | 32.0 | 37.0 | 19.0 | + | 1358.0 |
| +documentation  (+ kirjaaminen) | Noun | Keep | 0.360 | 11.0 | 54.0 | 10.0 | 36.0 | 20.0 | + | 120.0 |
| +different  (+ eri) | Noun | Keep | 0.356 | 33.0 | 39.0 | 30.0 | 34.0 | 21.0 | + | 121.0 |
| +challenge  (+ haste) | Noun | Keep | 0.370 | 17.0 | 41.0 | 16.0 | 33.0 | 22.0 | + | 364.0 |
| +do  (+ tehdä) | Verb | Keep | 0.358 | 37.0 | 37.0 | 33.0 | 33.0 | 22.0 | + | 1002.0 |
| +several  (+ usea) | Adj | Keep | 0.366 | 38.0 | 38.0 | 32.0 | 32.0 | 24.0 | + | 4826.0 |
| +can  (+ voi) | Noun | Keep | 0.389 | 41.0 | 41.0 | 30.0 | 30.0 | 25.0 | + | 247.0 |
| +new  (+ uusi) | Adj | Keep | 0.391 | 36.0 | 37.0 | 28.0 | 29.0 | 26.0 | + | 1108.0 |
| +get  (+ saada) | Verb | Keep | 0.405 | 34.0 | 34.0 | 28.0 | 28.0 | 27.0 | + | 26.0 |
| +unclear  (+ epäselvä) | Adj | Keep | 0.382 | 24.0 | 28.0 | 24.0 | 28.0 | 27.0 | + | 917.0 |
| +rush  (+ kiire) | Noun | Keep | 0.393 | 29.0 | 29.0 | 27.0 | 27.0 | 29.0 | + | 860.0 |
| +time  (+ aika) | Noun | Keep | 0.395 | 30.0 | 30.0 | 27.0 | 27.0 | 29.0 | + | 981.0 |
| +guideline  (+ ohje) | Noun | Keep | 0.405 | 27.0 | 36.0 | 24.0 | 27.0 | 29.0 | + | 1276.0 |
| +always  (aina) | Adv **) | Keep | 0.400 | 28.0 | 28.0 | 26.0 | 26.0 | 32.0 |  | 65.0 |
| +to document  (+ kirjata) | Verb | Keep | 0.419 | 32.0 | 34.0 | 23.0 | 25.0 | 33.0 | + | 519.0 |
| +mistake  (+ virhe) | Noun | Keep | 0.409 | 28.0 | 28.0 | 25.0 | 25.0 | 33.0 | + | 1855.0 |
| +issue  (+ asia) | Noun | Keep | 0.420 | 30.0 | 30.0 | 24.0 | 24.0 | 35.0 | + | 352.0 |
| +know  (+ tietää) | Verb | Keep | 0.423 | 25.0 | 25.0 | 23.0 | 23.0 | 36.0 | + | 3083.0 |
| +medication care  (+ lääkehoito) | Noun | Keep | 0.437 | 23.0 | 25.0 | 20.0 | 22.0 | 37.0 | + | 3999.0 |
| also  (myös) | Adv | Drop | 0.0 | 28.0 | 28.0 | 21.0 | 21.0 | 38.0 |  | 490.0 |
| +all  (+ kaikki) | Noun | Keep | 0.446 | 24.0 | 24.0 | 21.0 | 21.0 | 38.0 | + | 671.0 |
| +communication  (+ kommunikaatio) | Noun | Keep | 0.448 | 9.0 | 25.0 | 8.0 | 21.0 | 38.0 | + | 1354.0 |
| +system  (+ järjestelmä) | Noun | Keep | 0.450 | 22.0 | 22.0 | 20.0 | 20.0 | 41.0 | + | 43.0 |
| stay  (jää) | Noun | Keep | 0.450 | 22.0 | 22.0 | 20.0 | 20.0 | 41.0 |  | 505.0 |
| +part  (+ osa) | Noun | Keep | 0.447 | 18.0 | 21.0 | 17.0 | 20.0 | 41.0 | + | 1284.0 |
| +the same  (+ sama) | Noun | Keep | 0.450 | 22.0 | 22.0 | 20.0 | 20.0 | 41.0 | + | 3311.0 |
| +stay  (+ jäädä) | Verb | Keep | 0.462 | 21.0 | 22.0 | 18.0 | 19.0 | 45.0 | + | 61.0 |
| +may  (+ saattaa) | Verb | Keep | 0.467 | 19.0 | 19.0 | 18.0 | 18.0 | 46.0 | + | 433.0 |
| +use  (+ käyttö) | Noun | Keep | 0.464 | 17.0 | 18.0 | 17.0 | 18.0 | 46.0 | + | 618.0 |
| +digital  (+ sähköinen) | Adj | Keep | 0.484 | 21.0 | 21.0 | 18.0 | 18.0 | 46.0 | + | 1984.0 |
| +take  (+ ottaa) | Verb | Keep | 0.481 | 19.0 | 19.0 | 17.0 | 17.0 | 49.0 | + | 489.0 |
| +change/  amendment  (+ muutos) | Noun | Keep | 0.484 | 16.0 | 21.0 | 13.0 | 17.0 | 49.0 | + | 988.0 |

SAS Enterprise Text Miner 13.2. Node: Text filter. Terms treated as synonyms and unchecked terms. The analysis based on responses to open-ended question concerning medication communication challenges in hospital. Words and terms were translated from Finnish to English by the first author.

*) Adj=adjective **) Adv=adverb.
