# Supplementary material for: Healthcare professionals’ perceptions on medication communication challenges and solutions – text mining and manual content analysis - cross-sectional study
Source: BMC Health Serv Res. 2021 Nov 13;21:1226. doi: 10.1186/s12913-021-07227-0 (PMC8590289; doi:10.1186/s12913-021-07227-0)
Supplement: Supplementary file 3 — “Fifty most common terms linked to suggested medication communication enhancement in hospitals (N=195)”. Description of data: Medication communication enhancement terms extracted from free text of study data using IBM SPSS version 27 software for Windows (Chicago, IL, USA) in text filtering phase. [file 12913_2021_7227_MOESM3_ESM.docx]

Additional file 3

Fifty most common terms linked to suggested medication communication enhancement in hospitals (N= 195)

| **Term**  (original Finnish term in parentheses) | **Role** | **Status** | **Weight** | **Imported Frequency** | **Frequency** | **Number of imported Docs** | **#Docs** | **Rank** | **Parent/child Status** | **Parent ID** |
| --- | --- | --- | --- | --- | --- | --- | --- | --- | --- | --- |
| +be  (+ olla) | Verb | Alpha | Drop | 0.0 | 156.0 | 156.0 | 77.0 | 77.0 | 1.0 | + |
| +physician  (+ lääkäri) | Noun | Alpha | Keep | 0.259 | 59.0 | 64.0 | 48.0 | 52.0 | 2.0 | + |
| +patients  (+ potilas) | Noun | Alpha | Keep | 0.292 | 88.0 | 88.0 | 50.0 | 50.0 | 3.0 | + |
| +not  (+ ei) | Noun | Alpha | Keep | 0.287 | 49.0 | 80.0 | 30.0 | 49.0 | 4.0 | + |
| +medicine  (+ lääke) | Noun | Alpha | Keep | 0.317 | 92.0 | 95.0 | 46.0 | 48.0 | 5.0 | + |
| +all  (+ kaikki) | Noun | Alpha | Keep | 0.276 | 47.0 | 48.0 | 44.0 | 45.0 | 6.0 | + |
| +come  (+ tulla) | Verb | Alpha | Keep | 0.309 | 50.0 | 51.0 | 39.0 | 40.0 | 7.0 | + |
| +need/should  (+ pitää) | Verb | Alpha | Keep | 0.353 | 60.0 | 61.0 | 39.0 | 39.0 | 8.0 | + |
| +nurse  (+ hoitaja) | Noun | Alpha | Keep | 0.328 | 47.0 | 48.0 | 36.0 | 37.0 | 9.0 | + |
| +medication  (+ lääkitys) | Noun | Alpha | Keep | 0.355 | 47.0 | 48.0 | 33.0 | 34.0 | 10.0 | + |
| +prescription/order  (+ määräys) | Noun | Alpha | Keep | 0.357 | 40.0 | 42.0 | 31.0 | 32.0 | 11.0 | + |
| +do  (+ tehdä) | Verb | Alpha | Keep | 0.363 | 38.0 | 39.0 | 30.0 | 31.0 | 12.0 | + |
| +for example  (+ esim) | Noun | Alpha | Drop | 0.0 | 32.0 | 36.0 | 26.0 | 28.0 | 13.0 | + |
| also  (myös) | Adv *) | Alpha | Keep | 0.378 | 35.0 | 35.0 | 28.0 | 28.0 | 13.0 |  |
| always  (aina) | Adv | Alpha | Keep | 0.382 | 33.0 | 33.0 | 27.0 | 27.0 | 15.0 |  |
| +medication list  (+ lääkelista) | Noun | Alpha | Keep | 0.385 | 28.0 | 28.0 | 26.0 | 26.0 | 16.0 | + |
| +ICT system  (+ tietojärjestelmä) | Noun | Alpha | Keep | 0.403 | 24.0 | 24.0 | 23.0 | 23.0 | 17.0 | + |
| +guideline  (+ ohje) | Noun | Alpha | Keep | 0.439 | 22.0 | 23.0 | 20.0 | 20.0 | 18.0 | + |
| +medication prescription  (+ lääkemääräys) | Noun | Alpha | Keep | 0.430 | 20.0 | 21.0 | 19.0 | 20.0 | 18.0 | + |
| +information  (+ tieto) | Noun | Alpha | Keep | 0.496 | 30.0 | 30.0 | 19.0 | 19.0 | 20.0 | + |
| +give  (+ antaa) | Verb | Alpha | Keep | 0.456 | 25.0 | 26.0 | 19.0 | 19.0 | 20.0 | + |
| +issue  (+ asia) | Noun | Alpha | Keep | 0.458 | 23.0 | 23.0 | 19.0 | 19.0 | 20.0 | + |
| +can  (+ voi) | Noun | Alpha | Keep | 0.453 | 20.0 | 20.0 | 18.0 | 18.0 | 23.0 | + |
| +clear  (+ selkeä) | Adj **) | Alpha | Keep | 0.451 | 18.0 | 19.0 | 17.0 | 18.0 | 23.0 | + |
| +time  (+ aika) | Noun | Alpha | Keep | 0.453 | 20.0 | 20.0 | 18.0 | 18.0 | 23.0 | + |
| +one  (+ yksi) | Noun | Alpha | Keep | 0.458 | 17.0 | 17.0 | 17.0 | 17.0 | 26.0 | + |
| +to document  (+ kirjata) | Verb | Alpha | Keep | 0.467 | 20.0 | 20.0 | 17.0 | 17.0 | 26.0 | + |
| +medication care  (+ lääkehoito) | Noun | Alpha | Keep | 0.473 | 20.0 | 21.0 | 16.0 | 17.0 | 26.0 | + |
| +new  (+ uusi) | Adj | Alpha | Keep | 0.467 | 20.0 | 20.0 | 17.0 | 17.0 | 26.0 | + |
| +system  (+ järjestelmä) | Noun | Alpha | Keep | 0.473 | 17.0 | 17.0 | 16.0 | 16.0 | 30.0 | + |
| +the same  (+ sama) | Noun | Alpha | Keep | 0.469 | 15.0 | 16.0 | 15.0 | 16.0 | 30.0 | + |
| +patient health record system  (+potilastietojärjestelmä) | Noun | Alpha | Keep | 0.506 | 20.0 | 20.0 | 15.0 | 15.0 | 32.0 | + |
| +digital  (+ sähköinen) | Adj | Alpha | Keep | 0.499 | 15.0 | 15.0 | 14.0 | 14.0 | 33.0 | + |
| +get  (+ saada) | Verb | Alpha | Keep | 0.509 | 16.0 | 16.0 | 14.0 | 14.0 | 33.0 | + |
| +change/amendment  (+ muutos) | Noun | Alpha | Keep | 0.551 | 19.0 | 19.0 | 13.0 | 13.0 | 35.0 | + |
| +communication  (+ kommunikaatio) | Noun | Alpha | Keep | 0.514 | 13.0 | 14.0 | 13.0 | 13.0 | 35.0 | + |
| +additional  (+ lisä) | Noun | Alpha | Keep | 0.514 | 14.0 | 14.0 | 13.0 | 13.0 | 35.0 | + |
| +medication change  (+ lääkemuutos) | Noun | Alpha | Keep | 0.509 | 13.0 | 13.0 | 13.0 | 13.0 | 35.0 | + |
| +department  (+ osasto) | Noun | Alpha | Keep | 0.509 | 13.0 | 13.0 | 13.0 | 13.0 | 35.0 | + |
| +written  (+ kirjallinen) | Adj | Alpha | Keep | 0.530 | 13.0 | 13.0 | 12.0 | 12.0 | 40.0 | + |
| +oral  (+ suullinen) | Adj | Alpha | Keep | 0.530 | 13.0 | 13.0 | 12.0 | 12.0 | 40.0 | + |
| +more  (+ enemmän) | Adv | Alpha | Keep | 0.541 | 10.0 | 11.0 | 10.0 | 11.0 | 42.0 | + |
| +situation  (+ tilanne) | Noun | Alpha | Keep | 0.550 | 13.0 | 13.0 | 11.0 | 11.0 | 42.0 | + |
| +after  (+ jälki) | Noun | Alpha | Keep | 0.547 | 12.0 | 12.0 | 11.0 | 11.0 | 42.0 | + |
| +work  (+ työ) | Noun | Alpha | Keep | 0.550 | 13.0 | 13.0 | 11.0 | 11.0 | 42.0 | + |
| +check  (+ tarkistaa) | Verb | Alpha | Keep | 0.565 | 10.0 | 11.0 | 9.0 | 10.0 | 46.0 | + |
| +location  (+ Paikka) | Noun | Alpha | Keep | 0.559 | 10.0 | 10.0 | 10.0 | 10.0 | 46.0 | + |
| +place  (+ kohta) | Noun | Alpha | Keep | 0.559 | 10.0 | 10.0 | 10.0 | 10.0 | 46.0 | + |
| +different  (+ eri) | Noun | Alpha | Keep | 0.569 | 11.0 | 12.0 | 10.0 | 10.0 | 46.0 | + |
| +reporting  (+ raportointi) | Noun | Alpha | Keep | 0.565 | 10.0 | 11.0 | 9.0 | 10.0 | 46.0 | + |

SAS Enterprise Text Miner 13.2. Node: Text filter. Terms treated as synonyms and unchecked terms. The analysis based on responses to open-ended question concerning the suggestions for communication enhancement in hospital. Words and terms were translated from Finnish to English by the first author. *) Adv=adverb **) Adj=adjective
